# Impact of frequency of denture cleaning on microbial and clinical parameters – a bench to chairside approach

**DOI:** 10.1080/20002297.2018.1538437

**Published:** 2018-10-29

**Authors:** Gordon Ramage, Lindsay O’Donnell, Leighann Sherry, Shauna Culshaw, Jeremy Bagg, Marta Czesnikiewicz-Guzik, Clare Brown, Debbie McKenzie, Laura Cross, Andrew MacInnes, David Bradshaw, Roshan Varghese, Paola Gomez Pereira, Anto Jose, Susmita Sanyal, Douglas Robertson

**Affiliations:** a School of Medicine, Dentistry and Nursing, College of Medical, Veterinary and Life Sciences, University of Glasgow, Glasgow, UK; b GSK Consumer Healthcare, Weybridge, UK; c Syneos Health, Pune, India

**Keywords:** Denture, plaque, microbiology, denture cleansing, oral microbiome, antimicrobials

## Abstract

**Objective**: Robust scientific and clinical evidence of how to appropriately manage denture plaque is lacking. This two-part study (i) developed an *in vitro* model of denture plaque removal, and (ii) assessed effectiveness of these approaches in a randomised clinical trial.

**Method**: (i) a complex denture plaque model was developed using the dominant microbial genera from a recent microbiome analyses. Biofilms formed on polymethylmethacrylate were brushed daily with a wet toothbrush, then either treated daily for 5 days or only on Days 1 and 5 with Polident® denture cleanser tablets (3 min soaking). Quantitative and qualitative microbiological assessments were performed. (ii), an examiner-blind, randomised, crossover study of complete maxillary denture wearers was performed (n = 19). Either once-daily for 7 days or on Day 7 only, participants soaked dentures for 15 min using Corega® denture cleansing tables, then brushed. Denture plaque microbiological assessment used sterilized filter paper discs.

**Results**: The *in vitro* model showed daily cleaning with denture cleanser plus brushing significantly reduced microbial numbers compared to intermittent denture cleaning with daily brushing (p < 0.001). The clinical component of the study showed a statistically significant reduction in denture plaque microbial numbers in favour of daily versus weekly treatment (aerobic bacteria p = 0.0144). Both *in vitro* and *in vivo* studies showed that denture plaque biofilm composition were affected by different treatment arms.

**Conclusions**: This study demonstrated that daily denture cleansing regimens are superior to intermittent denture cleansing, and that cleansing regimens can induce denture plaque compositional changes. Clinicaltrials.gov registration: NCT02780661.

## Introduction

As the elderly population expands to a predicted two billion by 2050, the number of denture wearers will continue to rise. Edentulousness (loss of all teeth) is an irreversible clinical condition that can be described as an ultimate marker of oral disease burden [1]. Currently, around 20% of the UK population wear removable dentures of some form, with 70% of UK adults older than 75 years old wearing dentures [2]. Denture wearing is also associated with socioeconomic deprivation and is more common in women [3]. Many of these individuals have oral diseases related to their denture wearing including denture-induced stomatitis (DS), an inflammation of the denture bearing mucosa [4]. Poor oral hygiene is frequently observed within this group and several factors can impact the onset of DS such as salivary pH, smoking, sugar consumption, oral *Candida*, age of denture, and, importantly, denture cleanliness [5].

The high prevalence of edentulousness and associated DS highlight the importance of having consistent effective denture care regimens which patients can follow with confidence. However, the clinical and laboratory evidence to support one regime over another is not yet clear. To date, two systematic reviews featuring a total of six randomised controlled trials (RCTs) have been published [6,7]. These reviews concluded that there was lack of evidence on which to base guidelines and that further RCTs are required. Recent guidelines based on the available evidence suggest that removal of ‘bacterial biofilm’ is of paramount importance to sustaining good oral and systemic health and preventing DS [8]. These guidelines also advocate the reduction and maintenance of low levels of microbial denture plaque through daily soaking and/or brushing with an effective, non-abrasive cleanser, but there is a lack of clarity as to how this is best achieved. Subsequent meta-analyses indicate that in addition to existing methods, antiseptic mouthwashes, disinfection agents, natural antimicrobial substances, photodynamic therapy and microwave disinfection could all be effective adjunctive strategies for the management of denture hygiene [9].

Dentures are colonised when placed in the mouth by a complex microbial plaque biofilm, which contains numerous species of bacteria and fungi [10,11]. Plaque development and microbial retention are aided and enhanced by the irregular topographical surface including cracks and crevices which can exist within denture acrylic surfaces [12]. This environment also provides protection from chemotherapeutic agents and mechanical disruption methods, meaning that some denture surfaces can carry up to 10^11^ microbes per milligram of plaque [13,14]. Denture plaque biofilm also represents a reservoir for potential opportunistic respiratory pathogens [15].

There is a lack of consensus around suitable cleaning agents, with many denture wearers opting to use toothpaste to mechanically clean their dentures. However, this has been shown to induce abrasions, resulting in physical defects on the denture acrylic that may lead to enhanced microbial adhesion through altered surface topography [14,16–18]. Guidance on the frequency of cleansing is also lacking, although laboratory and clinical studies report that the sporadic use of denture cleansers facilitates the build-up of mature denture plaque biofilms [19–21]. Many chemotherapeutic interventions recommended are effective against planktonic oral bacteria, but unfortunately live intact biofilms are able to persist even after treatment with sodium hypochlorite [22]. These studies taken collectively suggest that denture cleansing is important, but more difficult to achieve than previously thought.

To shape and design an effective clinical trial, appropriate laboratory models are needed to assess *in vitro* the effect of novel approaches to denture cleansing on the biofilm. Unfortunately, progress here has been hampered by the fact that many denture plaque treatment studies have focussed on *Candida albicans*, primarily due to its role in denture-related disease [4, 21–25]. Available data conservatively estimates that at least 10-fold more bacteria than yeasts colonise the surface of dentures [10], clearly indicating that denture plaque has a polymicrobial and interkingdom composition [11]. Denture plaque biofilm models, such as a recently described 11 species interkingdom model, are likely to be more representative of the polymicrobial nature of the clinical situation [26]. In this study, we sought to adopt a bench-to-chairside approach to test the appropriateness of routine daily denture cleansing methods compared to intermittent methodologies. For this study we have used denture cleanser tablets that are based on generating hydrogen peroxide and peracetic acid. Due to the chronology of the entire study two different brand names were used (Corega and Polident) based on the countries in which they are marketed, and these products were used as per pack instructions and were chosen to represent the lower end of soaking practises that consumers typically use.

## Material and methods

### In vitro denture cleansing study

A denture plaque cleansing study and quantitative analysis of remaining viable cells was performed as previously described [26]. It was the aim to investigate whether a sequential denture cleansing technique was more advantageous than one treatment over the course of a 5-day treatment regimen. Briefly, laboratory strains were used to create a polymicrobial denture plaque biofilm model based on the most dominant genera/species identified from our recent denture microbiome study [10]. Polymethylmethacrylate (PMMA) discs were manufactured as described [27], providing the physical substrates on which biofilms were formed. The biofilms included *Streptococcus mitis* NCTC 12,261, *Streptococcus intermedius* ATCC 27,335, *Streptococcus oralis* ATCC 35,037, *C. albicans* 3153A, *Actinomyces naeslundii* ATCC 19,039, *Veillonella dispar* ATCC 27,335, *Rothia dentocariosa* DSMZ 43,762, *Lactobacillus casei* DSMZ 20,011 and *Lactobacillus zeae* DSMZ 20,178. Initially, *S. mitis, S. intermedius, S. oralis* and *C. albicans* were grown and standardised in artificial saliva to 1 × 10^7^ cells/mL. These were added to each well of a 24 well plate (Corning Inc, New York, USA) containing 13 mm^2^ PMMA discs (Chaperlin and Jacobs Ltd, Southend-on-Sea, UK) and incubated aerobically at 37°C for 24 h. Next, standardised (1 × 10^7^ cells/mL) *A. naeslundii, V. dispar, R. dentocariosa, L. casei* and *L. zeae* were added to the preformed 24-h biofilm and incubated at 37°C in 5% CO_2_ conditions for a further 4 days. Spent supernatants were removed and replaced with fresh artificial saliva daily.

**Figure 1.**
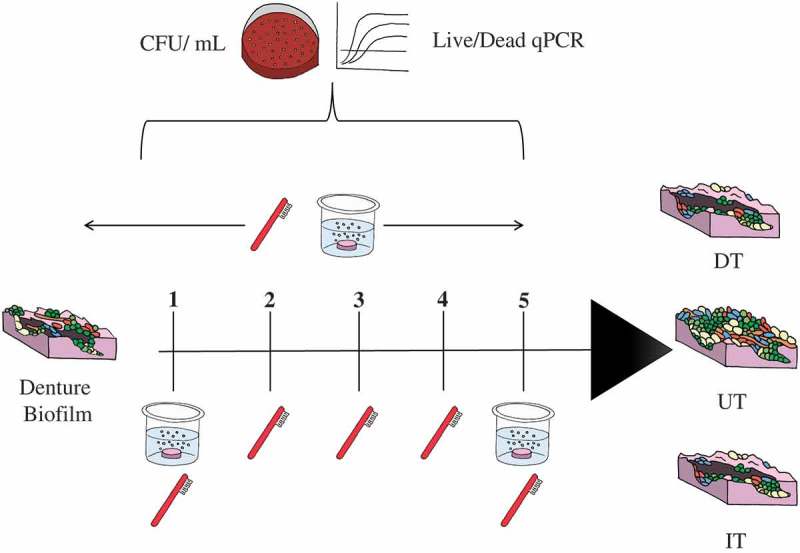
Sequential treatment of denture biofilm protocol.

Treatment regimens were either combinational daily treatment of brushing with hard water, followed by a daily 3 min soaking with a denture cleanser (Polident®3 min denture cleanser; GSK Consumer Healthcare, Weybridge, UK) (DC) for 5 consecutive days (DT group), daily brushing with hard water intermittent treatment (IT group) with DC on Day 1 and Day 5 only, or they were left untreated and were maintained in hard water corresponding to each treatment arm, serving as positive controls (UT group). Figure 1 shows a schematic of the treatment regimens.

Following each treatment, PMMA discs were incubated in Dey-Engley neutralising broth (Sigma-Aldrich, Gillingham, UK) for 15 min. PMMA discs were then sonicated in 1 mL phosphate-buffered saline (Sigma-Aldrich, Gillingham, UK) at 35 kHz for 10 min to remove the biomass, as previously described [21]. For quantitative analysis, both colony-forming unit (CFU) and quantitative live/dead PCR were performed, as described previously [26].

For the former, 20 μL denture plaque sonicate was transferred to a fresh microcentrifuge tube and serial log_10_ dilutions were performed in phosphate buffered saline, then 20 μL of each serial dilution was plated in triplicate on brain heart infusion + 10% blood agar plates (E&O Laboratories, Bonnybridge, UK), which were incubated aerobically and anaerobically at 37°C for 48 h [28]. The samples were also plated on Sabouraud dextrose agar and incubated at 30°C for 48 h for yeasts. Following incubation, the number of colonies was counted and represented as total viable aerobes, anaerobes and yeasts.

Viability of the treated biofilms was also assessed using live/dead PCR to enumerate the definitive and relative composition of the biofilms, a technique that has been shown to differentiate viable and dead cells from multispecies oral bacteria biofilm models. Samples were prepared as previously described, with some modifications [26]. In brief, 50 μM propidium monoazide (PMA) was added to each sonicated sample and incubated in the dark for 10 min to allow uptake of the dye. Samples were then exposed to a 650 W halogen light for 5 min before DNA was extracted using the QIAamp DNA mini kit, as per manufacturer’s instructions (Qiagen, Crawley, UK). No PMA controls were included for each sample to determine total biomass. The primers used were previously published and are listed in Table 1. Three independent replicates from each parameter were analysed in triplicate using a MxProP Quantitative PCR machine and MxPro 3000P software (Stratagene, Amsterdam, Netherlands). Samples were quantified to calculate the colony-forming equivalent (CFE) based upon a previously established standard curve methodology of bacterial CFU ranging from 1 × 10^3^ to 10^8^ CFU/mL [15]. Melting curve analysis was performed for all primer sets to ensure a single peak, which was indicative of primer specificity.

**Table 1.** Primer sequences for denture biofilm species.

Target	Primer sequence (5ʹ-3ʹ)	Reference
*16S*	**F** – CGCTAGTAATCGTGGATCAGAATG **R** – TGTGACGGGCGGTGTGTA	[26]
*18S*	**F** – CTCGTAGTTGAACCTTGGGC **R** – GGCCTGCTTTGAACACTCTA	[26]
*Streptococcus* spp.	**F** – GATACATAGCCGACCTGAG **R –** CCATTGCCGAAGATTCC	[52]
*A. naeslundii*	**F** – GGCTGCGATACCGTGAGG **R** – TCTGCGATTACTAGCGACTCC	[52]
*R. denticariosa*	**F** – GGGTTGTAAACCTCTGTTAGCATC **R** – CGTACCCACTGCAAAACCAG	(53)
*V. dispar*	**F** – CCGTGATGGGATGGAAACTGC **R** – CCTTCGCCACTGGTGTTCTTC	[52]
*L. casei*	**F –** TGCACTGAGATTCGACTTAA **R** – CCCACTGCTGCCTCCCGTAGGAGT	(54)
*L. zeae*	**F** – TGCATCGTGATTCAACTTAA **R –** CCCACTGCTGCCTCCCGTAGGAGT	(54)

### Data analysis

Data distribution, graph production and statistical analysis were performed using GraphPad Prism (version 5; La Jolla, CA, USA). After assessing whether data conformed to a normal distribution, One-way Analysis of Variance (ANOVA) and *t* tests were used to investigate significant differences between independent groups of data that approximated to a Gaussian distribution. A Bonferroni correction was applied to the p value to account for multiple comparisons of the data. Non-parametric data were analysed using the Mann-Whitney U-test or the Kruskal–Wallis test with a Dunn’s post-test to assess differences between independent sample groups. Statistical significance was achieved if p < 0.05. Principal Component Analysis (PCA) of the log (n) of CFUs of bacterial and yeast growth and CFEs of live bacteria and yeast after treatment were performed with R using in-built functions. Clustering (three clusters) was performed using the partitioning around mediods (pam) algorithm, a more robust version of k-means clustering, using the R package ‘cluster’. Visualisation by the package ‘ggplot2ʹ was utilised to provide figures.

### In vivo denture cleansing study

To assess the impact of daily or weekly DC on denture microbial count, composition, plaque and stain accumulation, a clinical trial was designed and carried out. This was a single-centre, randomised, controlled, examiner- and analyst-blind, crossover study conducted at Glasgow Dental Hospital and School, UK. The protocol was approved by an Independent Ethics Committee (West of Scotland Research Ethics Committee 3; Ref:16/WS/0092) and the study was conducted in accordance with the Declaration of Helsinki, the International Conference on Harmonisation of Technical Requirements for Registration of Pharmaceuticals for Human Use and local laws and regulations. All participants provided written informed consent prior to screening, demonstrated understanding of the protocol and were considered willing, able and likely to comply with all study procedures. This study was registered at ClinicalTrials.gov: NCT02780661. There was one amendment to the protocol to widen inclusion criteria to aid recruitment, this was not predicted to influence study outcomes.

### Participants

Participants in general good health, aged between 18 and 84 years inclusive, were recruited through self-referral and identification at treatment clinics at Glasgow Dental Hospital and School. Participants were required to have a completely edentulous maxillary arch restored with a conventional, full acrylic based, complete denture. The mandibular arch could be dentate, partial or full edentulous and could be restored with a stable complete, partial or implant supported denture. Maxillary dentures needed to be of a well-made design and construction, as assessed by the study examiner and moderately well-fitting at the screening visit according to the Kapur Index [29], Olshan Modification [30]: retention score > 2, stability score > 2. Exclusion criteria included pregnancy; breastfeeding; known/suspected intolerance or hypersensitivity to study materials or ingredients; a serious, severe or unstable medical condition that would make the participant unlikely to fully complete the study; an implanted cardiac pacemaker; taking a daily dose of medication that might interfere with the participant’s ability to perform the study or might affect efficacy assessments. Specific dental exclusion criteria included: a clinically significant or relevant oral abnormality that, in the investigator’s opinion, could affect study participation; recent (within 30 days) gingival/oral surgery.

### Study design and treatment

**Figure 2.**
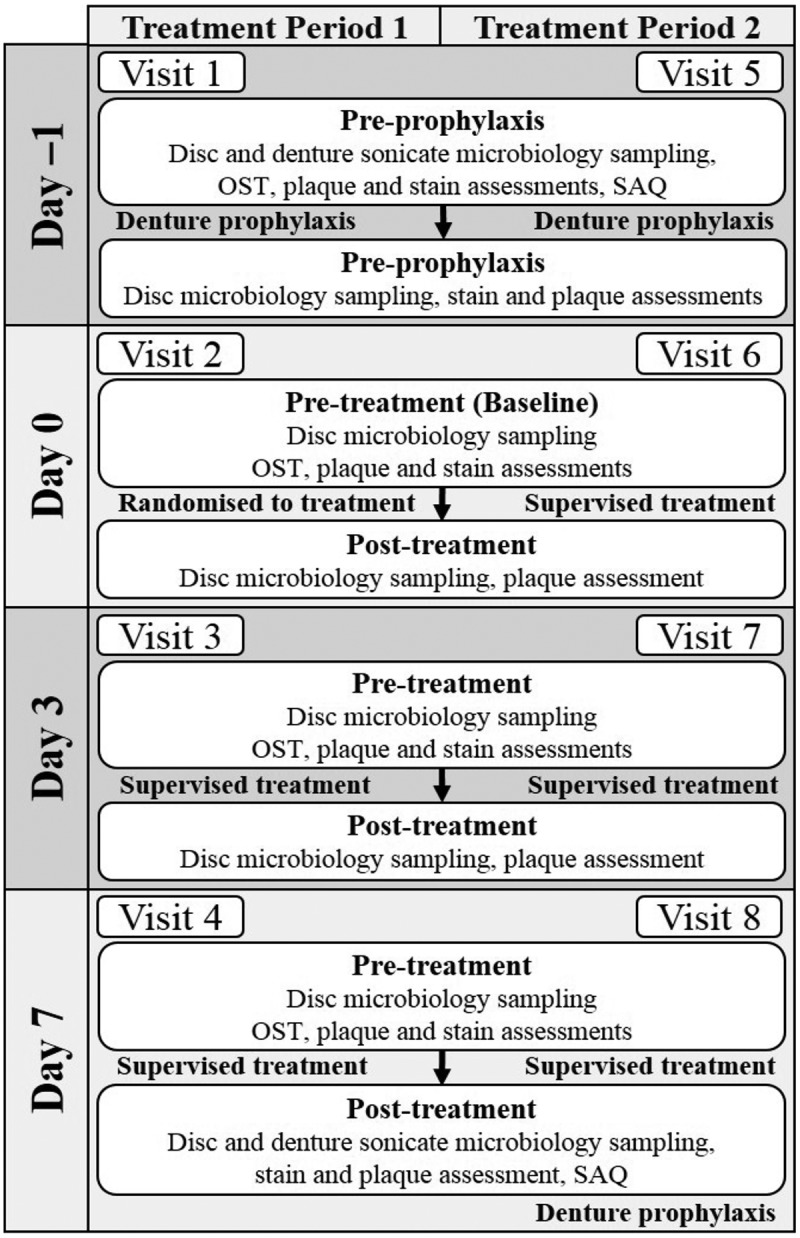
Study flow.

Study flow is detailed in Figure 2. At screening, participants provided written informed consent and eligibility was assessed. They received a dental prophylaxis and a denture prophylaxis of the maxillary complete denture; zero plaque and stain scores were confirmed by post-prophylaxis assessments. At the first study visit (Day 0) participants were assigned to a study treatment sequence order (1:1) in accordance with the randomisation schedule provided by the Biostatistics Department of GSK Consumer Healthcare. Randomisation numbers were assigned in ascending numerical order as each participant was determined to be fully eligible and consented for inclusion. All participants used supplied alkaline peroxide-based denture cleansing tablets (Corega® Tabs Dental Weiss für Racher [Denture Whitening for Smokers], German marketed product). The Daily Use group used one tablet per day (with supervised use at the site on Days 0, 3 and 7). The Weekly Use group used one tablet on Day 7 (supervised use at site on Day 7).

Dentures were soaked in a cup of 150 mL warm water with a cleanser tablet for 15 min, brushed for 30 s using the solution, then rinsed under running water for 10 s. At home, the Daily Use group repeated this procedure for the upper denture in the evening while the Weekly Use group carried out the procedure in warm water only. Both groups left dentures to soak overnight in 150 mL water. The lower denture, if present, was cleaned as usual but separately from the upper denture. Cleaning of the upper denture was not permitted in the morning; the lower denture could be cleaned as usual. Participants returned on Days 3 and 7. Following a washout period (7 ± 3 days) participants returned for treatment period 2, as described above including an initial denture and dental prophylaxis. Participants refrained from smoking, including e-cigarettes and the use of chewing tobacco or other tobacco products for the duration of the study. They could not use any other denture cleaners or regimens to clean their upper dentures. Participants were asked not to use denture fixative, xylitol-containing or oral-care type chewing-gum for the duration of the trial as these could impact hygiene parameters.

Examiners (clinical and laboratory scientists) and data analysts were blinded to treatment allocation. Examiners were not allowed to be in the room where test products were stored or allocated. Additionally, dispensing staff were not involved in any effectiveness assessments. Examiners were calibrated prior to commencement of the trial.

### Microbial sampling

**Figure 3.**
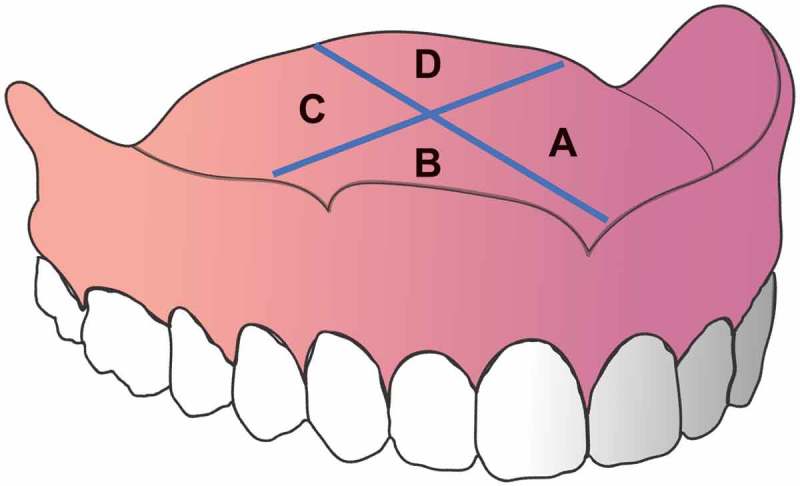
Quadrants used for sampling.

To collect samples from the tissue-fitting surface of the maxillary denture for microbiological analysis, the denture was sampled in four quadrants lateral to the midline and corresponding to the palatal rugae (Figure 3). Pre-prophylaxis and pre-treatment samples were taken from the left rough (A) and left smooth (D) denture side. Post prophylaxis and post-treatment samples were taken from the right rough (B) and right smooth (C) denture surface.

A pre-sterilized 10 mm filter paper disc (Sigma-Aldrich, Gillingham, UK) was lightly pressed against the allocated quadrant for 20 s prior to aseptic removal. Two discs, of the allocated quadrants (depending on the time point), were pooled and processed appropriately for microbiology cultures (CFU/disc) to assess microbial counts (aerobic bacteria, anerobic bacteria and *C. albicans)*, and for qPCR analysis to assess denture microbial composition, as described previously [26]. To investigate the microbial counts and microbial composition on the whole maxillary denture, on Day 1 (pre-prophylaxis) and Day 7 (post-treatment) the maxillary denture was placed in a sterile bag with 50 mL PBS and placed in a sonic bath for 15 min at 35 kHz. This procedure removed microbes adhered to all areas of the denture. The resulting samples were processed appropriately for microbiology cultures (CFU/denture) and for molecular microbial analysis by qPCR (CFE/denture), as described previously [26].

### Clinical assessments

Denture plaque was assessed on three areas separately: fitting surfaces, polished surfaces and denture teeth (facial/buccal and palatal) based on the modification of the Clinical Categorization of Denture Cleanliness Index [31], where 0 = No visible plaque; no matter adherent to the side of the dental probe on light scraping; 1 = No visible plaque; matter adherent to the side of the dental probe on light scraping; 2 = Deposits of plaque just visible on careful examination without need to confirm by scraping; 3 = Deposits of plaque ‘clearly visible’; 4 = Gross plaque deposits (‘velvet appearance’). For a given denture area under examination, the highest score of that area was recorded.

Denture stain was also assessed on these areas, by modification of the Denture Cleanser Index [32]. The stain scale was related to the percentage of the surface covered in stain where 0 = No staining detectable; 1 = Little staining (<25% of surface stained); 2 = Moderate staining of surface (25–50% of surface stained); 3 = Severe staining of surface (>50% of surface stained).

### Safety

Adverse events (AEs) were collected from the start of the denture and dental prophylaxis at the Screening Visit until 5 days following last administration of the study product. Incidents were documented from the Baseline visit (Visit 2). Oral soft tissue examinations were performed at all baseline visits pre-prophylaxis or pre-treatment. The safety population was defined as all participants who were randomised and received at least one dose of study treatment during the study.

### Evaluation criteria and data analysis

As variation and treatment effect were unknown, a formal sample size calculation was not possible. A total of 17 participants was determined to be suitable to assess effectiveness and safety of treatment products. The primary population for efficacy assessment was the intent-to-treat (ITT) population, defined as all participants who were randomised, received at least one dose of study treatment and provided at least one post-baseline assessment of microbial count from disc sampling. The per protocol (PP) population was defined as all participants in the ITT population who had at least one assessment of efficacy considered unaffected by protocol violations.

The primary objective was to evaluate and compare change from baseline in microbial count from denture disc samples of the Daily Use and Weekly Use groups on Day 7, with comparison on Day 3 as a secondary objective. Exploratory objectives included evaluation and comparison of the denture sonicate microbial count on Day 7 and, on Days 3 and 7 plaque levels, microbial composition from disc samples and stain levels on the maxillary denture. All endpoints were tested under the general hypotheses of a treatment difference between Daily and Weekly product use. All statistical analyses were conducted using Statistical Analysis System (SAS) version 9.2.

The three microbial counts (aerobic bacteria microbial count, anaerobic bacteria microbial count and *Candida* microbial count) were analysed separately for both treatment regimens at Days 3 and 7 compared to pre-treatment Day 0. Microbial counts were log transformed (base 10) prior to any analysis being performed. To be able to analyse all samples, if no microbes were retrieved (‘0ʹ values), a constant (+1) was added to all values prior to log transformation. Changes from baseline were analysed using an analysis of covariance (ANCOVA) model with treatment and period as fixed effects, participant-level (mean across treatment periods) and period-level pre-treatment (on Day 0) baseline scores (with the same transformation) as covariates. To allow model estimates to be representative of the studied population, participant was included in the model as a random effect. Confidence Intervals (CIs) and p-values were calculated for the difference between the treatments (Daily use vs Weekly use). Model assumptions were investigated, no assumptions were violated.

Microbial counts at Day 7 and denture plaque levels at Days 3 and 7 compared to Day 0 pre-treatment were calculated with each endpoint using an ANCOVA as above. Model assumptions were investigated, no assumptions were violated. Microbial composition at Days 3 and 7, assessed by qPCR from disc samples, was represented for both treatments in a stacked bar chart as percentage of each microbial group. The sum of the eight oral microbial groups analysed was considered as 100%. Denture stain levels at Days 3 and 7 on tissue fitting surfaces, polished surfaces and denture teeth stain score were analysed separately as a change from baseline to compare between treatment regimens (Daily use and Weekly use).

### Examiner repeatability

At each visit, for a random sample of participants, stain and plaque assessments were repeated by the examiner to check consistency in measuring plaque and stain levels on the denture surfaces. For each parameter of stain and plaque assessments, a Fleiss-Cohen weighted kappa coefficient (κ), along with the 95% CI was calculated for the repeatability analysis for each denture surface (tissue fitting surfaces, polished surfaces, denture teeth). Reliability was deemed excellent if κ>0.75; fair to good if 0.4≤κ≤0.75; poor if κ<0.4

## Results

### Quantitative analysis of in vitro denture plaque biofilm

**Figure 4.**
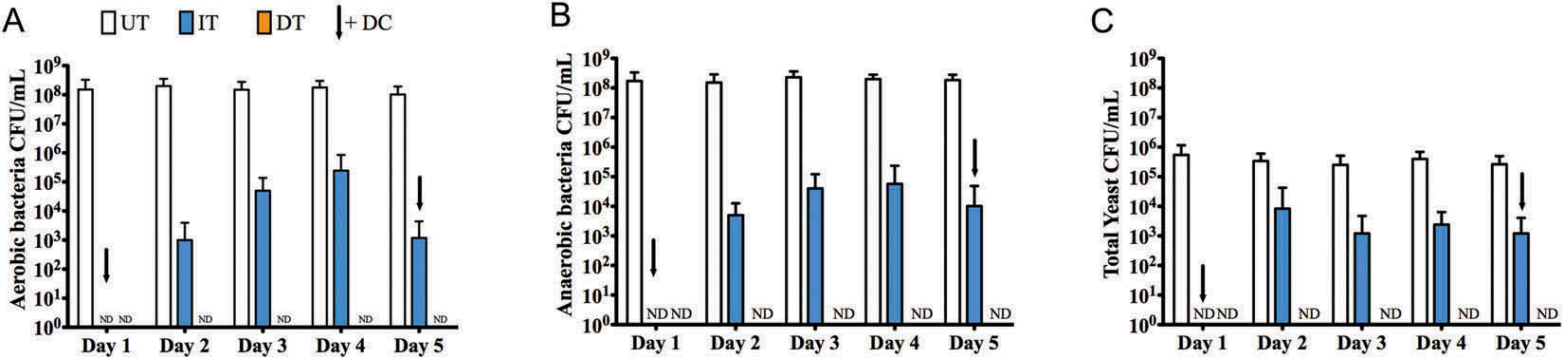
Daily CFU/mL counts of A) aerobic bacteria, B) anaerobic bacteria and C) total yeast count (±standard deviation) post treatment.

**Figure 5.**
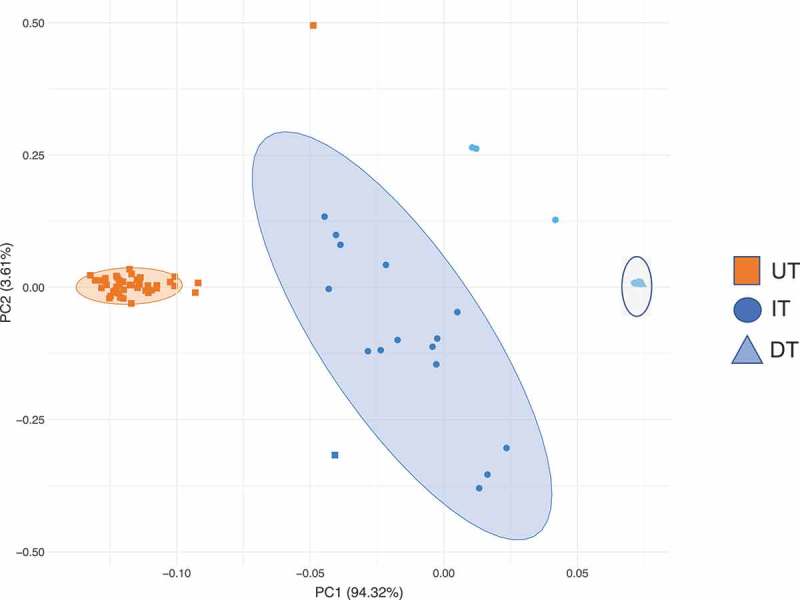
Principal component analysis showing different *in vitro* treatment outcomes associated with total and viable cell populations.

The *in vitro* analysis of different denture treatment regimens on multispecies denture plaque biofilms was carried out over the course of 5 days. Three groups were included: untreated biofilms as a positive control (UT), daily brushing followed by denture cleansing (DT) and intermittent denture cleansing (IT). Total aerobes, anaerobes and yeasts were initially quantified using CFU analysis over 5 days (Figure 4). For the DT group, no viable CFUs were detectable (ND) on any day for aerobes, anaerobes and yeasts, whereas for the IT group, viable bacteria were detected on Days 2, 3, 4 and 5 (approximately 10^3^ to 10^5^ CFU/mL), and on Days 2, 3 and 4 for yeasts (approximately 10^3^ to 10^4^ CFU/mL). No significant changes in overall microbial levels were observed in this time frame for the UT group, with consistent levels of 10^8^ and 10^6^ CFU/mL detected for bacteria and yeasts, respectively (data not shown). Both the DT and IT groups showed a statistically significant reduction in CFU’s for aerobes, anaerobes and yeasts (p < 0.001) compared to the UT group, though DT was consistently and statistically significantly more effective than IT (p < 0.001). To visually illustrate the overall effects of the treatment regimens, a PCA analyses was performed (Figure 5). Clustering demonstrated three independent clusters between the treatments as highlighted by the ellipses and colored clusters. PC1 and PC2 are displayed with PC1 displaying over 94% of the variation between samples, the variation along this component distinguishes between the treatment types.

**Figure 6.**
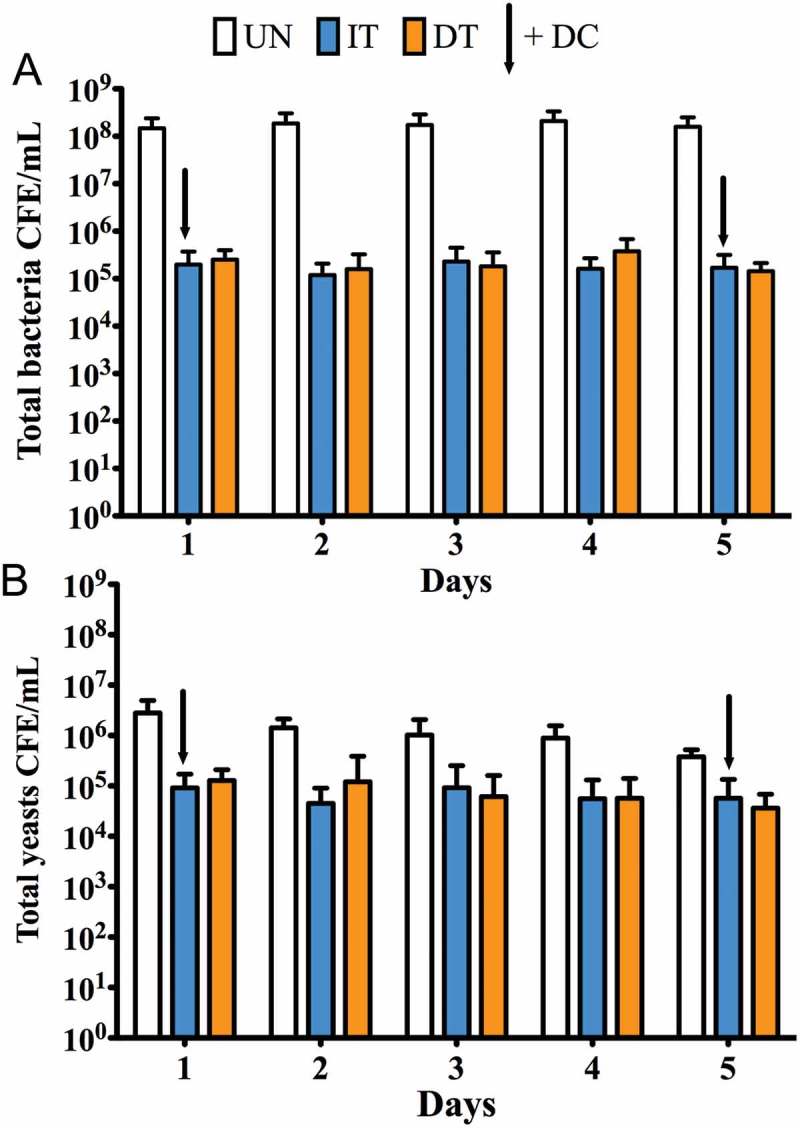
Daily CFE counts of (a) total bacteria and (b) total yeasts post treatment (±standard deviation).

Given that total viable cell counting is prone to inaccuracy (e.g. clumps of cells), and the possibility for the carry-over of actives, despite neutralisation, then a quantitative PCR was employed as an adjunctive assay to supplement these observations. For total bacteria retained on the PMMA surface following treatment (Figure 6), the number of bacteria was significantly lower in both the IT and DT groups compared to the untreated control by approximately 3 log_10_ (p < 0.001), though no discernible differences were observed in retained bacteria between these groups (data not shown). The number of yeasts isolated from the discs shows a similar pattern to that of the bacteria, in that by comparison with the UT group there were significantly fewer yeast cells in both the DT and IT groups, with an approximately 1 log_10_ reduction (p < 0.001), though again there were no differences between DT and IT groups. Live qPCR analysis was also performed to assess how many of the retained cells were viable, based on whether cell membranes were compromised or not. This showed that despite these treatments, approximately 1 × 10^4^ and 1 × 10^3^ CFE/mL of bacteria and yeasts, respectively, remained viable through Days 1 to 5, irrespective of treatment group (data not shown).

**Figure 7.**
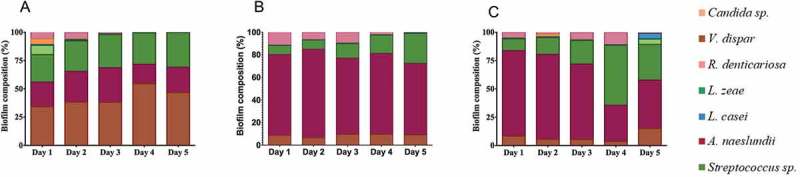
Microbial composition as assessed by qPCR from *in vitro* denture disc samples. (a) Untreated [UT], (b) Denture cleanser [DT], (c) Brushing [IT].

To assess whether any of the treatment regimens impacted the composition of the denture plaque biofilms, changes in the individual species contribution to each biofilm were investigated over the 5-day time course. The total cell count was quantified and converted into a proportion of the overall biofilm to determine the contribution of each species. Interestingly, it was observed that the UT group showed a number of changes over the 5 days, with *V. dispar, A. naeslundii* and *Streptococcus* species dominating the biofilm, with a notable increase in *V. dispar* at Days 4 and 5 (Figure 7(a)). Reciprocally, a reduction in *R. dentocariosa, C. albicans, L. zeae* and *L. casei*, was observed daily as the biofilms matured. The DT group showed a biofilm dominated by *A. naeslundii*, with increasing proportions of *Streptococcus* species (Figure 7(b)), though the IT group was initially dominated by *A. naeslundii*, followed by *Streptococcus* species (Figure 7(c)). Overall, these data showed that different interventions have the capacity to alter denture plaque composition, in a treatment dependent manner.

### Quantitative analysis of in vivo denture plaque biofilm

#### Clinical

A total of 25 participants were screened, with a total of 19 participants randomized to a treatment sequence. There were no participant withdrawals with all 19 randomized participants completing the study. There was one protocol violation in the Daily use regimen and one in the Weekly use regimen treatment periods, which led to data exclusion. Of the 19 participants the majority were female (n = 12, 63.2%) and white (n = 19, 100%), with a mean age of 68.7 years (SD 5.10, range 60–75 years). At baseline (Day 0, pre-treatment), the transformed microbial counts [Log_10_(Count+1)] were slightly higher in the Weekly use treatment at 2.27 Log_10_(CFU/disc) compared to 2.04 for Daily use for aerobic bacteria. These values for anaerobic bacteria were 2.47 and 2.07, respectively.

#### Microbial counts

**Figure 8.**
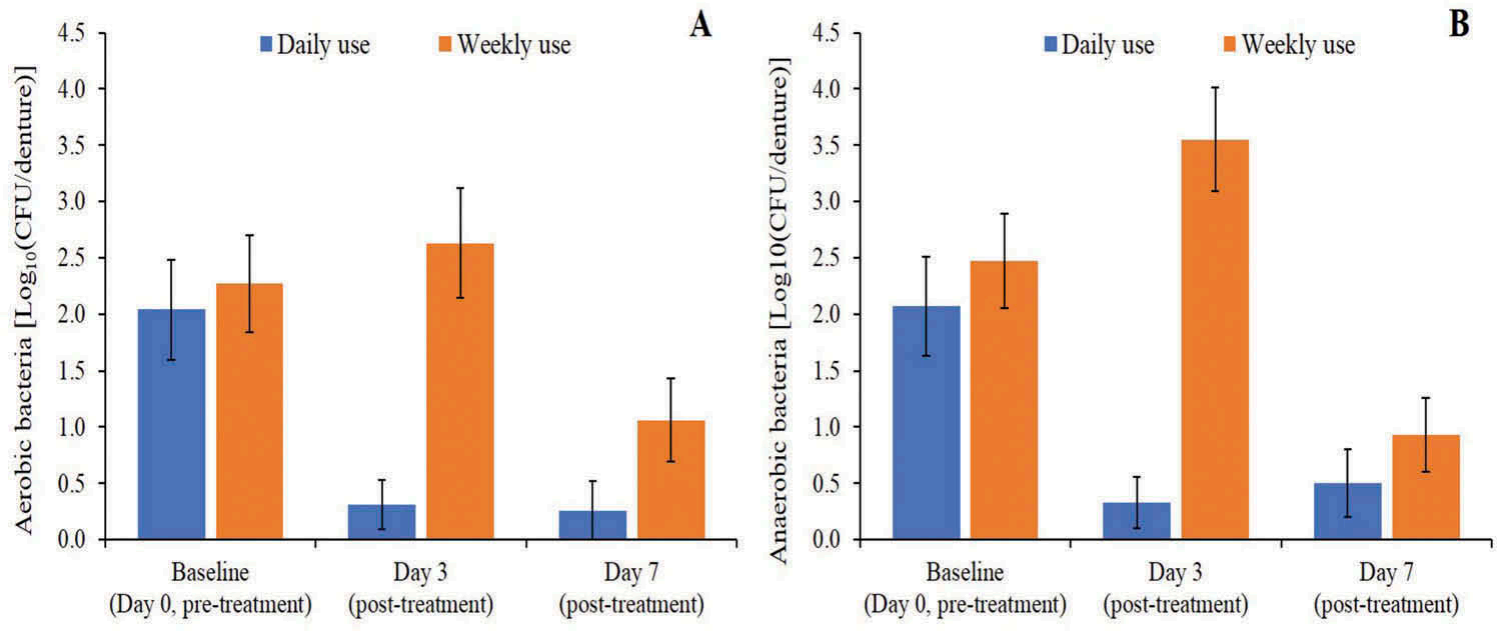
(a) Aerobic bacteria and (b) Anaerobic bacteria microbial count Log_10_ (CFU/disc) by visit and treatment from denture disc samples (±standard error) (ITT population).

A reduction from baseline in both aerobic and anaerobic bacteria in the denture disc samples was observed on Day 7 for both treatments, resulting in values of adjusted mean values of 0.26 Log_10_(CFU/disc) and 1.06 Log_10_(CFU/disc) aerobic bacteria in the Daily Use and the Weekly use treatment respectively, and 0.50 Log_10_(CFU/disc) and 0.93 Log_10_(CFU/disc) anaerobic bacteria for each treatment, respectively (Figure 8). A statistically significant difference between treatments was observed for aerobic bacteria microbial count at Day 7, with a greater reduction observed for the Daily use treatment (Table 2) (−0.86 adjusted mean treatment difference, p-value 0.0144). For the anerobic bacteria, a treatment difference was observed in favour of the Daily use treatment; however, this difference was not statistically significant (−0.48 adjusted mean treatment difference, p-value 0.1879). *C. albicans* cultured from disc samples were mostly zero and were therefore not further analysed (data not shown).

**Table 2.** Statistical analysis of change from baseline in aerobic bacteria and anaerobic bacteria microbial counts (CFU/disc) from denture disc (DDisc) and denture sonicate (DSon) samples (ITT Population).

	Change from baseline		Treatment comparison		
	Daily use Mean (SE)	Weekly use Mean (SE)	Difference (95% CI)	p-value	Ratio (95% CI)
**Aerobic bacteria (post treatment)**					
DDisc Day 3	−1.85 (0.39)	0.47 (0.39)	−2.32 (−3.43, −1.20)	0.0002	0.005 (0.0004, 0.0628)
DDisc Day 7	−1.92 (0.32)	−1.06 (0.32)	−0.86 (−1.53, −0.19)	0.0144	0.137 (0.0295, 0.6369)
DSon Day 7	−0.36 (0.13)	−0.48 (0.13)	0.12 (−0.257, 0.505)	0.5118	1.33 (0.55, 3.20)
**Anaerobic bacteria (post treatment)**					
DDisc Day 3	−1.94 (0.38)	1.28 (0.38)	−3.22 (−4.24, −2.19)	<.0001	0.001 (0.0001, 0.0064)
DDisc Day 7	−1.80 (0.33)	−1.31 (0.32)	−0.48 (−1.23, 0.26)	0.1879	0.328 (0.0589, 1.8257)
DSon Day 7	−0.35 (0.13)	−0.42 (0.14)	0.07 (−0.33, 0.46)	0.7316	1.17 (0.47, 2.88)
***C. albicans* (post treatment)**					
DSon Day 7	−0.69 (0.44)	−0.08 (0.45)	−0.60 (−1.86, 0.66)	0.3250	0.25 (0.01, 4.53)

Difference is first named treatment minus second named treatment such that a negative difference favours the first named

At Day 3, a reduction from baseline in aerobic and anaerobic bacteria was observed for the Daily use treatment, resulting in adjusted mean values of 0.31 and 0.33 Log_10_(CFU/disc) respectively. Subjects in the Weekly use treatment, who did not use the denture cleanser tablet but cleaned their denture with water instead, showed an increase in both microbial counts. Aerobic bacteria increased to 2.63 Log_10_(CFU/disc) while anaerobic bacteria increased to 3.55 Log_10_(CFU/disc). A statistically significant between treatment difference was observed for aerobic and anaerobic bacteria microbial count at Day 3 with the greater reduction observed for the Daily use treatment (−2.32 adjusted mean treatment difference, p-value 0.0002 for aerobic bacteria and −3.22, p-value <0.0001 for anaerobic bacteria). Following the Daily use treatment regimen, a high proportion of subjects had no microbial counts at Day 3 (17 out of 19 for both aerobic and anaerobic bacteria) while in the Weekly use treatment regimen no microbes were retrieved from fewer subjects (seven out of 19 subjects for aerobic bacteria and four out of 19 for anaerobic bacteria).

With regards to exploratory objective, denture sonicate microbial samples were collected only at Day −1 visit (Screening visit) before initial prophylaxis (pre-prophylaxis) and at Day 7 (post-treatment). No statistically significant between treatment difference was observed for aerobic or anaerobic bacteria microbial count at Day 7 in the denture sonicate samples (Table 2). *C. albicans* microbial count from both time points and treatments were retrieved at lower numbers than aerobic or anerobic bacteria. A between treatment difference was observed in favour of the Daily use treatment; however, this difference was not statistically significant (−0.60 adjusted mean treatment difference, p-value 0.325).

#### Microbial composition

**Figure 9.**
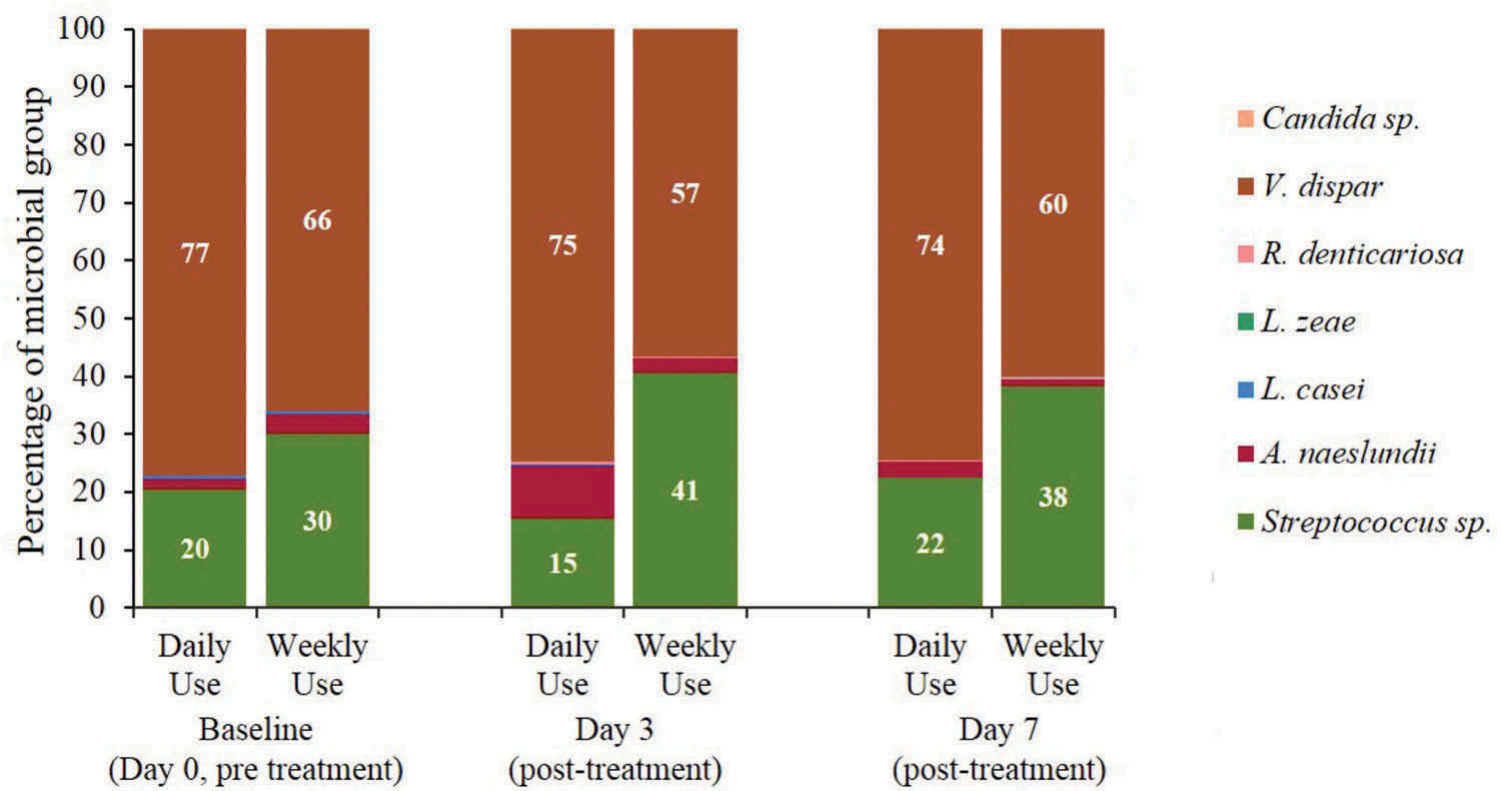
Microbial composition as assessed by qPCR from denture disc samples (ITT population).

Microbial composition by treatment and visit was assessed using specific primers targeting bacteria known to be associated with dentures, as described above. A stacked bar chart is presented for their relative abundance, considering as a 100% the sum of the eight microbial groups targeted (Figure 9). There was no formal statistical analysis performed for the microbial composition data. A dominance of *V. dispar* and *Streptococcus* species was observed at the different time points and for both treatments (ranging between 57 and 77% and 15 and 41%, respectively). *A. naeslundii* was detected with both treatments and visits but in lower relative abundance (ranging between 1 and 9%). *R. dentocariosa, L. casei* and *Candida* species were only minor contributors to the microbial composition of the dentures (in the disc samples), detected at less than 1%. *L. zeae* was not detected in any of the samples. In the Weekly use treatment group, a slight increase in *Streptococcus* species was detected from Baseline (pre-treatment) to Day 3 and Day 7 (post-treatment) and a slight decrease in *V. dispar* and other minor groups. However, no overall evident changes in microbial composition were observed either for the Daily use or the Weekly use treatment, with both having a dominance of *V. dispar* and *Streptococcus* species.

#### Denture plaque and stain scores

**Figure 10.**
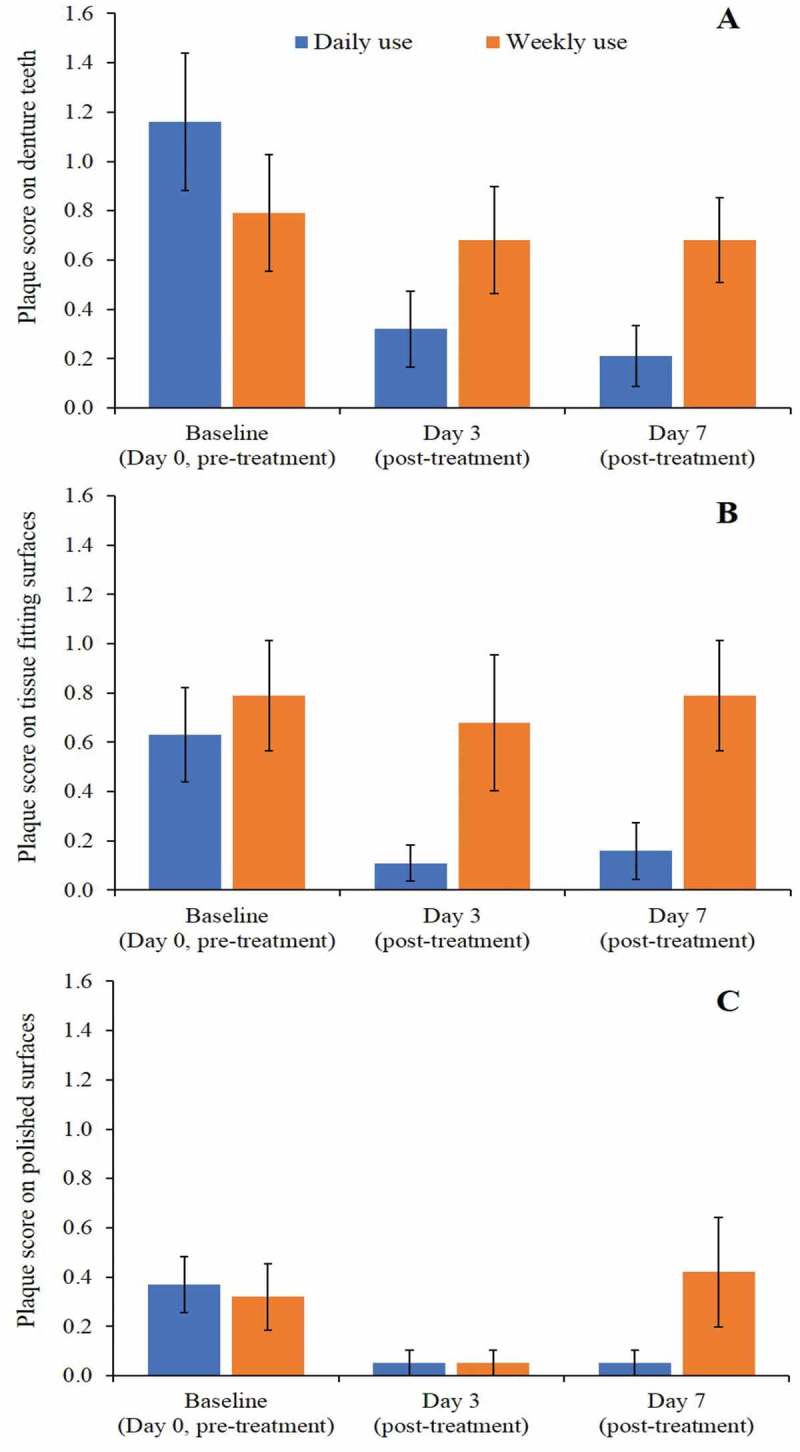
Raw means plaque score on (a) Denture teeth, (b) Tissue fitting surfaces and (c) Polished surfaces (±standard error) (ITT population).

Overall, low plaque scores were observed on all the denture surfaces, particularly on the polished surfaces (Table 3). Examiner repeatability for plaque was excellent with a weighted kappa of 0.968 [95% CI 0.922,1.00]. Despite the overall low plaque scores, a decrease following Daily use of the denture cleanser was observed at Days 3 and 7, while the Weekly use treatment regimen led to small changes depending on Day and denture area examined (Figure 10). At Day 3 there were differences observed in favor of the Daily use treatment for the three surfaces; however, these differences were not statistically significant. At Day 7, a statistically significant between treatment difference was observed for denture teeth and for tissue fitting surfaces with the greater reduction observed for the Daily use treatment in both surfaces (−0.54 adjusted mean treatment difference, p-value 0.0211 for denture teeth; −0.57, p-value 0.0320 for tissue fitting surface)

**Table 3.** Statistical analysis of change from baseline in plaque score in denture teeth, tissue fitting surfaces and polished surfaces (ITT Population).

	Change from baseline			Treatment comparison
	Daily use Mean (SE)	Weekly use Mean (SE)	Difference (95% CI)	p-value
**Denture teeth (post treatment)**				
Day 3	−0.65 (0.20)	−0.30 (0.20)	−0.35 (−0.92, 0.23)	0.2307
Day 7	−0.79 (0.15)	−0.26 (0.15)	−0.54 (−1.99, −0.09)	0.0211
**Tissue fitting surfaces (post treatment)**				
Day 3	−0.58 (0.20)	−0.05 (0.20)	−0.52 (−1.11, 0.07)	0.0765
Day 7	0.52 (0.18)	0.05 (0.18)	−0.57 (−1.095, −0.05)	0.0320
**Polished surfaces (post treatment)**				
Day 3	−0.30 (0.05)	−0.28 (0.05)	−0.02 (−0.15, 0.12)	0.7875
Day 7	−0.30 (0.16)	0.09 (0.16)	−0.39 (−0.86, 0.07)	0.0953

Difference is first named treatment minus second named treatment such that a negative difference favours the first named

Stain levels were very low throughout the study, particularly at baseline where most of the surfaces had a ‘no staining detectable’ score. Overall, at Day 7 for the three surfaces there was a difference observed in favour of the Daily use treatment. However, these differences were very small and not statistically significant for any of the three surfaces (data not shown).

#### Safety results

Overall, 28 treatment emergent AEs (TEAEs) were reported by 13 (68.4%) participants, 14 in each of the treatment groups. There were 21 oral TEAEs reported by 12 participants (63.2%) (10 in the Daily Use and 11 in the Weekly Use groups). None of the TEAEs were considered related to treatment. All TEAEs were mild or moderate in nature and all but ‘Lip injury’ had resolved by study end. There were no serious AEs or incidents reported and no participants withdrew from the study due to AEs. The use of the denture cleanser tablet was shown to be generally well tolerated in the study.

## Discussion

While there is limited evidence on how denture care should be implemented [6,7], the guidelines are clearer on what should not be done [8]. Cleaning in boiling water and storing the dentures dry should be avoided to minimize physical warping. The storage solution should be changed frequently to prevent microbial overgrowth within the water. Prolonged exposure to sodium hypochlorite/bleach containing products should also be avoided due to its detrimental impact on denture materials, particularly metals [33]. The use of microwave disinfection in combination with denture cleansers and brushing has also been shown to disinfect dentures *in vivo* [34], though microwaves may also physically distort denture acrylics [35]. A number of unconventional approaches to denture care, including soaking in vinegar, baking soda, sodium chloride (table salt) and liquid soaps, were identified in a recent study [36]. Many of these remedies are lacking in either efficacy and/or material compatibility [33]. Nevertheless, without proper professional advice many denture wearers may well continue these alternative practices.

Collectively the current evidence we have, which in many instances remains weak, highlights the need for improved denture cleansing techniques capable of dealing with a range of bacteria, in addition to highly tolerant candidal cells. Disruption of the denture biofilm is also critical to improving oral health. This may be achieved either through mechanical means such as a brush or using sonic cleaning devices which may be a more effective method of cleansing and decolonising dentures. Alternative strategies may include chemicals and enzymes capable of digesting and disaggregating biofilms, which could allow improved penetration and activity of agents in cracks, crevices and pores. On current evidence, mechanical disruption coupled with effective antimicrobial agents is likely to be the most desirable option.

PMMA is the primary denture material of choice, though this has an uneven surface that results in a heterogenous topography that yeasts and bacteria can co-colonise, forming biofilms and escaping from denture cleansing therapies [21,37,38]. Investigations to determine optimal methods for cleaning dentures have focussed on the various physical and chemical cleansing techniques, both individually and in combination. However, most of these investigations evaluate treatment over a short period of time and therefore do not accurately simulate an optimal denture routine clinically [8,39,40]. Daily denture cleansing treatment of *C. albicans* biofilms has been previously investigated, with results indicating that despite a significant reduction in viable *C. albicans* cells, a residual reservoir of yeast cells remained, indicative of ineffective cleansing [21,41,42]. A limitation of these studies was the use of a single species biofilm model, as this is not reflective of the polymicrobial denture environment. The present study aimed to address this by developing a more complex model that would allow assessment of repeated, longitudinal treatments as well as physical and chemical treatment modes.

Quantitative analysis of different denture treatment regimens on multispecies *in vitro* denture plaque biofilms was carried out over the course of 5 days. Results indicated that regular daily cleaning provided a significantly greater benefit than intermittent cleaning, even with the use of brushing together with the chemical disinfection. The results showed that despite active treatments of denture material, significant quantities of microorganisms were retained on the PMMA surface, that could only be released by sonication. Thus, although a considerable number of microbes remained on the discs post cleansing, the treatments employed were significantly effective given the extensive reduction in the overall microbial burden. Live qPCR results suggest that the denture plaque biofilm may contain the dormant persister cell phenotype that are unaffected by treatments, but cannot necessarily be cultured. Overall, these data suggest that denture biofilm composition is dependent on whether and how the biofilms are treated, which largely agrees with our previous studies [21,26]. However, this model benefits from its inception and design based on the first reported denture plaque microbiome [10]. This has facilitated us to use this as a robust first line screening tool capable of discerning quantitative differences in denture plaque biofilms in a reproducible manner.

The clinical study also followed a similar pattern with regard to the microbial count reduction between daily vs intermittent cleaning. Several studies have demonstrated the ability of alkaline peroxide based denture cleansers on reducing denture plaque biofilm [43,44]. The current study is first of its kind evaluating the impact of daily vs intermittent (once weekly cleaning) using alkaline peroxide based denture cleanser tablets.

A statistically significant greater reduction in aerobic bacteria microbial count was reported for the Daily use treatment regimen at Day 7 and in aerobic and anaerobic bacteria at Day 3 in comparison with the Weekly use treatment regimen. At Day 3 the aerobic and anaerobic microbial count in the Weekly use treatment was higher than at Day 0, indicating that the microbial biofilm grew from baseline when participants cleaned their dentures daily with water. At Day 7, where participants in both treatment regimens used the denture cleanser tablet, the aerobic and anaerobic bacterial count was reduced from baseline in both treatments. The result at Day 3 presumably reflects the antimicrobial activity delivered from the denture cleanser tablet, in comparison with water. However, the differences observed at Day 7 are intriguing since both groups had received identical treatments immediately prior to sampling. The data suggest that the biofilm developed in the weekly treatment group was more resistant to a single treatment with denture cleanser in comparison to the biofilms developed in the daily treatment group. This is in accordance with the results seen in the *in vitro* element of the present study, and in many other studies of mature biofilms from oral and other sources. It provides a microbial line of evidence to support previous clinical studies that have suggested regular cleaning of dentures to be beneficial to overall oral health [6,7]. Soaking alone in a denture cleanser may not be sufficient for adequate plaque removal [45], and is in line with the widely held belief around mechanical cleaning methods being important for physical plaque removal. Some studies have demonstrated an additional benefit linked to use of denture cleanser tablets on plaque removal compared to brushing alone. Sheen and colleagues demonstrated use of alkaline peroxide based cleanser resulted in 42% reduction (p = 0.0014) in plaque levels after 2 weeks of use compared to brushing with water [46].

Despite the relatively low denture plaque scores throughout this study, a statistically significantly greater reduction in denture teeth and tissue fitting surfaces plaque scores were found for the group using the Daily use treatment regimen compared to the Weekly use treatment at Day 7. Nishi et al (2012) collected denture plaque from denture wearers and analysed the effect of denture brush use, cleansing frequency and cleaning solution [47]. They concluded that the use of brush was associated with lower amounts of microbes and that, unsurprisingly, daily use was better than monthly use. In the general population they did not find a difference between daily and 3–4 times per week, but in those patients who were in nursing homes daily cleaning was the most effective. It is possible that these most vulnerable patients are unable to clean sufficiently themselves and our study would confirm that daily cleaning is advisable. Our results are also in agreement with the small clinical study carried out by Sheen et al (2000), which showed that denture plaque levels could be reduced using a daily brushing technique, but that the addition of an active cleanser reduced the rate of plaque formation and was more effective than water and brushing alone [46]. Moreover, results of the present study correlate with the conclusion of Kiesow et al (2016), who reported that specialist denture cleanser tablets provide a good combination of microbial efficacy, while also maintaining material compatibility [33]. The use of denture cleansers was also shown to lead to a significant reduction of microbial burden compared to a mouthwash [48]. This study, alongside the preceding evidence, has in part addressed the inadequacies in the literature that was concluded from previous systematic reviews [6,7]. Indeed, these data provide greater evidence that frequent use of denture cleansers is an effective strategy for supporting a low microbial bioburden that logically will maintain mucosal health. To further define and evolve our understanding of mucosal health we have developed techniques to investigate and evaluate microbial population dynamics. This approach may have translational benefits for improving existing denture cleansers developed to target specific groups of pathogenic denture plaque microorganisms.

The denture microbial composition was investigated from the disc samples by a qPCR targeted approach at Days 0, 3 and 7. Eight microbial groups were selected based on findings from a previous microbiome high throughput sequencing study of denture wearers [10]. In the present study a relative dominance of *V. dispar* and *Streptococcus* species was observed in both treatment groups and at all time points, with other microbial groups contributing in smaller proportions. No apparent difference between treatments were observed. *Streptococcus* and *Veillonella* have been documented as early colonizers and dominant microbes in healthy oral biofilms [49–51] and have been reported as major components of dentures in participants without stomatitis [10,52]. *Actinobacteria* spp. have been reported as abundant components of the denture’s microbiome [10]; however, in this study they were present in a relative low abundance (<10%). This study would benefit from a full microbiome analyses, though whether these data would add value in terms of driving evidence for the best treatment regimens remains to be seen. Our qPCR approach provides an intermediate and more economical approach to assessing changes in microbial dynamics.

## Conclusions

The present study shows how basic science understanding has enabled the development of an *in vitro* denture plaque model system that mimics the development of plaque on dentures in the mouth. Treatment with denture cleansers on a regular, daily basis in both an *in vitro* model and in a clinical study of denture wearers was more effective in reducing microbial numbers and plaque scores in comparison with intermittent treatments.
